# Editorial: Nutrient supplementation and its impact on pregnancy outcomes

**DOI:** 10.3389/fnut.2023.1357893

**Published:** 2024-01-04

**Authors:** Debora F. B. Leite, Renato T. Souza, Juliana Y. Enos

**Affiliations:** ^1^Department of Gynecology and Obstetrics, Federal University of Pernambuco, Recife, Brazil; ^2^Department of Gynecology and Obstetrics, University of Campinas, Campinas, Brazil; ^3^College of Health Sciences, Noguchi Memorial Institute for Medical Research, University of Ghana, Accra, Ghana

**Keywords:** iron, folic acid, vitamin D, omega 3, supplementation, high risk pregnancy, low risk pregnancy, antenatal care

It is now widely known that pregnancy is a window of opportunity for life-long health, both for the mother and her offspring. Besides the increasing metabolic demands, it is also a critical anabolic period for the fetus' development and growth. Thus, other than supporting a healthy diet and stimulating exercise, nutritional supplementation has been associated with improved pregnancy outcomes. However, the mechanisms for the effective and efficient delivery of nutritional supplements to pregnant women remain limited, and whether adverse outcomes can be predicted or prevented through active surveillance of the levels of specific nutrients is controversial. This Research Topic collects eight articles dedicated to providing evidence on nutritional requirements and supplementation in pregnancy and their impact on perinatal outcomes.

Three out of the eight manuscripts address iron and folic acid (IFA). This is relevant since iron–folate deficiency during pregnancy is a major public health problem globally, occurring in both developed (high-income) and developing countries (1). Even in well-nourished women, IFA dietary intake is insufficient in pregnancy. Consequently, the World Health Organization (2) recommends the supplementation of all pregnant women with iron (30–60 mg/day) and folic acid (400 mcg/day), due to their role in preventing maternal anemia, preterm birth, and reducing the incidence of low birth weight (1).

Unfortunately, universal coverage of IFA supplementation remains a challenge. Several contributory factors have been identified. The results of Bora et al. and Delie et al., in India and Ethiopia, respectively, show that maternal adherence to IFA supplementation is not homogenous, and this may be a major contributor to the poor uptake and effectiveness of IFA supplementation. They have analyzed national registries on IFA distribution across each country over the years, not differentiating low-risk from high-risk pregnant women. In these populations, although there was a progressive increasing adherence to IFA supplementation, it was determined by wealth index, educational attainment, and socioeconomic status. On the other hand, dosing ferritin may be the most adequate strategy for diagnosing iron deficiency anemia in cases of thalassemia, as outlined by Wang et al.

IFA supplementation is of major importance. Apart from their role in oxygenizing tissues and promoting fetal growth, iron is also important in neurogenesis and myelination, and folic acid is involved with one-carbon transfers, DNA synthesis, and neural cell proliferation and differentiation, as highlighted by Heland et al. Hence, iron–folate deficiency during pregnancy may corroborate the effect of low socioeconomic status during childhood, which may affect neurodevelopment structure (3).

Another three manuscripts address adverse pregnancy outcomes. Preeclampsia and gestational diabetes mellitus (GDM) are two of the most important and intriguing adverse pregnancy outcomes. Preeclampsia is caused by impaired trophoblast invasion and systemic endothelial dysfunction early in pregnancy, leading to vasoconstriction and oxidative stress (4). Its prevention is complex, but it would benefit from nutrient supplementation in cases of low dietary intake. This is true for calcium (2), and there is room for investigating vitamin E. In a large cohort from China, first-trimester vitamin E levels between 7.3 and 11.4 mg/dL implicated the lowest risk for preeclampsia, whereas levels < 7.3 mg/dL were associated with the highest risk. In this case, Shi et al. consider that vitamin E supplementation for women with low vitamin E levels is beneficial and should be explored in different populations. Still, in the first trimester, folate levels can compose a prediction model for preeclampsia (Zhang Y. et al.).

GDM is intrinsically linked with maternal insulin resistance and fat metabolism physiologic changes. In current practice, screening and diagnosing GDM strategies throughout pregnancy are important and necessary although insufficient. Due to the rise in GDM prevalence in recent years (5), we should improve monitoring from the first trimester with novel biomarkers and technologies, such as dosing polyunsaturated fatty acids (PUFAs) and other lipid metabolites, according to Liu et al. Interestingly, PUFAs are also involved in better neurodevelopment outcomes and lower body and abdominal fat in childhood (Heland et al.). Moreover, whether equilibrating omega-6 and omega-3 fatty acids intake in pregnancy reduces GDM and ameliorates long-term outcomes should be further tested.

Finally, dosing nutrients might be important for women undergoing reproductive assisted technologies. Zhang L. et al. have demonstrated that higher folate levels are associated with improved oocyte competence and embryo quality in cases of *in vitro* fertilization (IVT).

In conclusion, nutrient supplementation is important for improving pregnancy outcomes for all categories of women. Maternal nutritional status and the levels of specific nutrients should be monitored by epidemiological and clinical approaches in various settings. The evidence gathered and presented in this Research Topic reinforces the importance of maternal nutrient supplementation in healthy pregnancy outcomes for both low- and high-income populations and contexts.

## Author contributions

DL: Conceptualization, Writing – original draft, Writing – review & editing. RS: Writing – original draft, Writing – review & editing. JE: Writing – original draft, Writing – review & editing.
